# The Added Value of Cerebral Imaging in Patients With Pyogenic Spinal Infection

**DOI:** 10.3389/fneur.2021.628256

**Published:** 2021-05-04

**Authors:** Bedjan Behmanesh, Florian Gessler, Johanna Kessel, Fee Keil, Volker Seifert, Markus Bruder, Matthias Setzer

**Affiliations:** ^1^Department of Neurosurgery, Goethe University Hospital, Frankfurt am Main, Germany; ^2^Department of Medicine, Infectious Diseases Unit, Goethe University Hospital, Frankfurt am Main, Germany; ^3^Institute of Neuroradiology, University Hospital Frankfurt, Goethe University Hospital, Frankfurt am Main, Germany

**Keywords:** spinal infection, brain infection, cerebral imaging, surgery, blood culture

## Abstract

**Background:** The incidence of pyogenic spinal infection has increased in recent years. In addition to treatment of the spinal infection, early diagnosis and therapy of coexisting infections, especially of secondary brain infection, are important. The aim of this study is to elucidate the added value of routine cerebral imaging in the management of these patients.

**Methods:** This was a retrospective single-center study. Cerebral imaging consisting of cerebral magnetic resonance imaging (cMRI) was performed to detect brain infection in patients with a primary pyogenic spinal infection.

**Results:** We analyzed a cohort of 61 patients undergoing cerebral imaging after diagnosis of primary pyogenic spinal infection. The mean age in this cohort was 68.7 years and the gender distribution consisted of 44 males and 17 females. Spinal epidural abscess was proven in 32 (52.4%) patients. Overall positive blood culture was obtained in 29 (47.5%) patients, infective endocarditis was detected in 23 (37.7%) patients and septic condition at admission was present in 12 (19.7%) Patients. Coexisting brain infection was detected in 2 (3.3%) patients. Both patients revealed clinical signs of severe sepsis, reduced level of consciousness (GCS score 3), were intubated, and died due to multi-organ failure.

**Conclusions:** Brain infection in patients with spinal infection is very rare. Of 61 patients with pyogenic spinal infection, two patients had signs of cerebral infection shown by imaging, both of whom were in a coma (GCS 3), and sepsis.

## Introduction

The incidence of spondylodiscitis in western countries has been rising over recent decades. The main reasons are demographic changes, extended life expectancy, and improved access to medical services and treatment of chronic diseases (1–6). Moreover, the median age of patients suffering from spondylodiscitis is increasing, and a high number of these patients harbor a significant number of comorbidities (6–8). The incidence of coexisting brain infections in these patients is still unclear. Until now there is a significant lack of scientific data reporting on this issue. Brain infections are relatively rare, but they are potentially serious and have a poor prognosis with reported overall mortality rates in patients hospitalized with central nervous system infections up to 32% (9–14). Therefore, early diagnosis and treatment of brain infection are important for a patient's outcome. The aim of the present study is to highlight the incidence of secondary brain infection, risk factors, and clinical outcomes in patients with pyogenic spondylodiscitis admitted to a university spine surgery unit. We further evaluate the value of cerebral imaging in the management of these patients.

## Methods and Materials

All patients admitted to the authors' institution with newly developed primary pyogenic spondylodiscitis had their information entered into an institutional database. Spinal MRI and CT scans were performed to confirm the diagnosis, extent of infection, and presence of bone destruction.

In collaboration with our colleagues from the Infectious Diseases Unit, cerebral imaging for detection of brain infection in patients with spinal infection was performed after confirmation of the spinal infection to elucidate the proportion of coexisting brain infection. All cases with initial brain infection and subsequent confirmation of spinal infection were excluded.

All patients underwent additional MRIs after admission to our department for treatment for spinal infection. Further treatment and diagnostic workup were discussed with a multidisciplinary focus. Results of cerebral imaging were interpreted by a senior neuroradiologist.

### Study Approval

This study was approved by the local ethics committee of the authors' institution.

### Statistical Analysis

Comparison of important baseline characteristics and clinical parameters between patients with and without coexisting brain infection was made using the Fisher exact test for categorical variables and the Wilcoxon-Mann-Whitney test for continuous variables. *P* < 0.05 (2-tailed) was deemed significant. All analyses were carried out with GraphPad Prism version 7.0 statistical software, La Jolla, USA.

## Results

From 2016 to 2019, 61 out of 183 patients with spinal infection underwent cerebral imaging for detection of any pathologies within the brain. The mean age in this cohort was 68.7 years. The cohort consisted of 44 males and 17 females. Among all patients undergoing cerebral imaging, two (3.3%) revealed a coexisting brain infection. Cerebritis with intraventricular empyema in one case and three brain abscesses in the other case were noted. The maximum diameter of the largest abscess was 2 × 3.5 cm, the remaining two were smaller (1 × 1.5 cm and 1 × 1.2 cm). Both patients were admitted in a severe septic condition, were treated in the ICU, and died due to multi-organ failure. The most likely etiology of the spinal and cerebral infection was sepsis. Detection of the pathogen was feasible in 42 (69%). Staphylococcus aureus was the most frequent infectious agent and was detected in 31 patients (74%), followed by Streptococcus in 6 (14%), Propionibacterium acnes and *E. coli* in 2 (5%) cases each, and Serratia marcescens in 1 patient. No prior surgery was performed on these two patients. The initial GCS scores in both patients were 3.

No other differences between patients with or without brain infection were found, especially upon analysis of the rate of spinal epidural abscess, the location of the infection, the number of affected levels, and the prevalence of positive blood culture. A septic condition upon admission and in-hospital mortality was significantly seen often in patients suffering from a spinal and coexisting brain infection. Detailed characteristics are shown in Table 1.

**Table 1:** Baseline characteristics of patients with spinal infection and brain infection.

**Patients' characteristics**	**Brain infection**	**No brain infection**	***P*-value**
No., *n* (%)	2 (3.3)	59 (96.7)	
Age	64.5	68.7	0.9
Sex, male, *n* (%)	2 (100)	42 (71.2)	1.0
Location			
Cervical, *n* (%)	1 (50)	8 (13.5)	0.3
Thoracic, *n* (%)	0	14 (23.7)	
lumbar, *n* (%)	1 (50)	37 (72.7)	0.5
Affected level			
1, *n* (%)	0	46 (78)	
2, *n* (%)	1 (50)	6 (10.2)	0.2
>2, *n* (%)	1 (50)	7 (11.9)	1.0
Spinal epidural abscess, *n* (%)	2 (100)	30 (50.8)	0.5
Positive blood culture, *n* (%)	2 (100)	27 (45.8)	0.2
Endocarditis, *n* (%)	0	23 (39)	
Diabetes, *n* (%)	0	9 (15.3)	
Sepsis, *n* (%)	2 (100)	10 (16.9)	0.04
HIV, *n* (%)	0	2 (3.4)	
Obesity, *n* (%)	0	14 (23.7)	
IDU, *n* (%)	0	1 (1.7)	
In hospital mortality, *n* (%)	2 (100)	4 (6.8)	0.008

**Figure 1:**
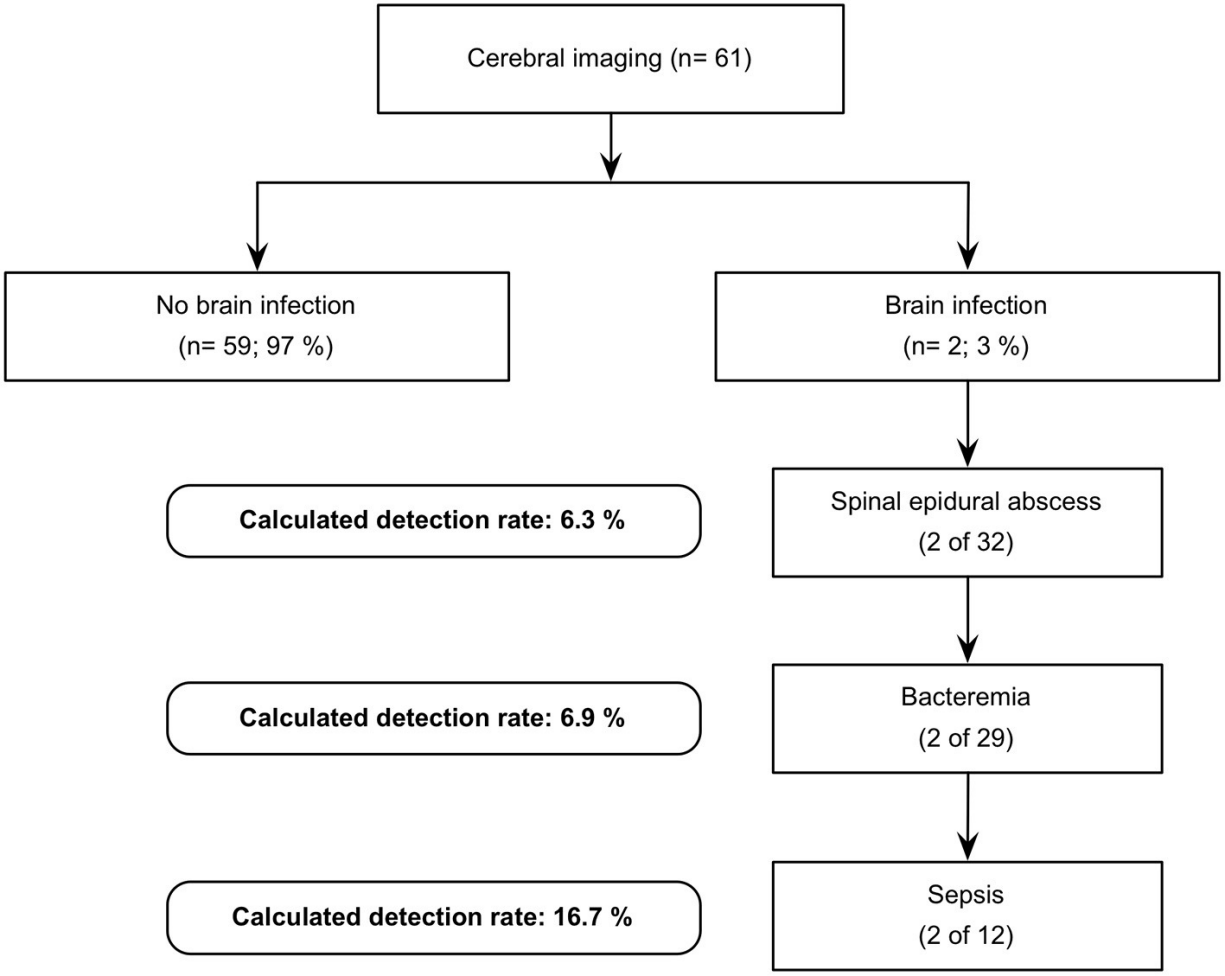
Flow diagram.

In view of the data we have collected, the expected detection rate of brain infection in patients with spinal infection increases with the following predictive factors: spinal epidural abscess (*n* = 32; 6.25%), positive blood culture (*n* = 29; 6.9%), and a septic condition upon admission (*n* = 12; 16.7%). Figure 1 illustrates a possible decision tree for performing cerebral imaging in order to detect brain infection as well.

## Discussion

Within this study, we have investigated the incidence of secondary brain infection among patients with pyogenic spondylodiscitis. Of 61 patients with pyogenic spinal infection, 2 patients had signs of cerebral infection by imaging, both of whom were in coma (GCS 3) and in sepsis, which would have prompted cerebral imaging. It should be noted that cerebral imaging would also be ordered in all patients with reduced consciousness, not just those with a pyogenic spinal infection. To the best of our knowledge, the routine diagnosis of brain infection in the management of patients with pyogenic spondylodiscitis has not been described until now.

Spinal infections are a growing problem that deserves our multidisciplinary attention from cardiologists, radiologists, microbiologists, infectiologists, and spinal surgeons. The spinal infection mostly represents a sign of a systemic infection, proven by bacteremia, a septic condition, fever, or neurologic deficit, which then manifest themselves as spinal and even cerebral infections (15–17). Therefore, it is crucial to detect coexisting or secondary complications that could be associated with it. Nonetheless, focusing on the best treatment of the spinal infection and neglecting coexisting or secondary brain infections may result in poor patient outcomes despite enormous efforts. Therefore, it is mandatory to establish multidisciplinary diagnostic and treatment algorithms for patients with spinal infections. One aspect that has not been highlighted is the incidence of brain infection.

Symptoms such as headache, confusion, poor mental status, and poor responsiveness are typical for cerebral infections and might be covered by the application of sedatives in patients with severe sepsis treated within an ICU (10, 12, 18, 19). Detecting brain infections is very important in these patients especially.

The prevalence of sepsis in our investigated series was the strongest predictive criterion that we found potentially associated with the incidence of brain infection. Furthermore, it seems that the mortality rate is increased when cerebral and spinal infections are harbored. Since an altered mental state is another common feature of sepsis and even severe pneumonia, it is not common to order brain MRIs in all cases. Here, we focus on this aspect and recommend brain imaging in patients with sepsis and altered mental status.

CNS infections, such as brain abscesses, epidural abscess, meningitis, and septic embolic stroke are often attributed to hematogenous spread, contiguous spread, recent neurosurgical procedures, or penetrating head trauma (2, 20). Endocarditis or pulmonary infections are the most common sources of hematogenous spread and are often associated, as coexisting infections, with pyogenic spinal infections (16, 21, 22). In many cases, the origin of the spinal infections might be a systemic infection, which causes sepsis, endocarditis, spinal infection, and brain infection. Nevertheless, all patients with spinal infections were initially introduced to our department for the treatment of spinal infections. Here, we want to highlight and postulate the need and advantage of a multidisciplinary approach, which results in a higher detection rate of coexisting infections and complications. Patients with severe sepsis and an altered mental state at our ICU revealed, besides the spinal infection, brain infection detected by routine cerebral imaging. Therefore, we recommend routine brain MRI especially in patients with signs of systemic infection, such as bacteremia, sepsis, or endocarditis and an altered mental state.

The strengths of the study include being the first mention of the incidence of concurrent brain infections in patients with spinal infections. Coexisting brain infection is most notable in patients who present with mental status changes. Brain MRI should be offered to such patients.

### Limitations

This a relatively small study of only 61 patients from only one medical facility. So generalizability of the findings is very limited. The conclusion of conducting CNS imaging in patients who present with spinal infection and mental state changes is not novel but is the standard of care in most cases.

## Conclusion

Brain infections in patients with spinal infections are very rare. Nevertheless, cerebral imaging should be performed in patients with altered mental states and sepsis. Spinal infection without sepsis did not result in cerebral infection.

## Data Availability Statement

The raw data supporting the conclusions of this article will be made available by the authors, without undue reservation.

## Ethics Statement

The studies involving human participants were reviewed and approved by Local ethics committee Goethe University. The patients/participants provided their written informed consent to participate in this study.

## Author Contributions

BB: study enrollment, data collection, writing, formatting manuscript, and submission. FG: data collection and interpretation of the data. JK and FK: data collection and follow-up examinations. VS: supporting and supervising the study. MB: data collection and spine surgeon. MS: supervising the study and senior surgeon. All authors contributed to the article and approved the submitted version.

## Conflict of Interest

The authors declare that the research was conducted in the absence of any commercial or financial relationships that could be construed as a potential conflict of interest.
